# Glucose absorption drives cystogenesis in a human organoid-on-chip model of polycystic kidney disease

**DOI:** 10.1038/s41467-022-35537-2

**Published:** 2022-12-23

**Authors:** Sienna R. Li, Ramila E. Gulieva, Louisa Helms, Nelly M. Cruz, Thomas Vincent, Hongxia Fu, Jonathan Himmelfarb, Benjamin S. Freedman

**Affiliations:** 1 Division of Nephrology, University of Washington School of Medicine, Seattle, WA 98109 USA; 2 Kidney Research Institute, University of Washington School of Medicine, Seattle, WA 98109 USA; 3 Institute for Stem Cell and Regenerative Medicine, University of Washington School of Medicine, Seattle, WA 98109 USA; 4 Department of Medicine, University of Washington School of Medicine, Seattle, WA 98109 USA; 5 Department of Laboratory Medicine & Pathology, University of Washington School of Medicine, Seattle, WA 98109 USA; 6 Department of Bioengineering, University of Washington, Seattle, WA 98109 USA; 7 Division of Hematology, University of Washington School of Medicine, Seattle, WA 98109 USA

**Keywords:** Polycystic kidney disease, Induced pluripotent stem cells, Molecular medicine, Apicobasal polarity

## Abstract

In polycystic kidney disease (PKD), fluid-filled cysts arise from tubules in kidneys and other organs. Human kidney organoids can reconstitute PKD cystogenesis in a genetically specific way, but the mechanisms underlying cystogenesis remain elusive. Here we show that subjecting organoids to fluid shear stress in a PKD-on-a-chip microphysiological system promotes cyst expansion via an absorptive rather than a secretory pathway. A diffusive static condition partially substitutes for fluid flow, implicating volume and solute concentration as key mediators of this effect. Surprisingly, cyst-lining epithelia in organoids polarize outwards towards the media, arguing against a secretory mechanism. Rather, cyst formation is driven by glucose transport into lumens of outwards-facing epithelia, which can be blocked pharmacologically. In PKD mice, glucose is imported through cysts into the renal interstitium, which detaches from tubules to license expansion. Thus, absorption can mediate PKD cyst growth in human organoids, with implications for disease mechanism and potential for therapy development.

## Abstract (web summary)

In polycystic kidney disease (PKD), fluid-filled cysts arise from tubules. Here the authors show that subjecting organoids to fluid shear stress in a PKD-on-a-chip microphysiological system promotes cyst expansion via an absorptive pathway.

## Introduction

Autosomal dominant polycystic kidney disease (PKD) is commonly inherited as a heterozygous, loss-of-function mutation in either *PKD1* or *PKD2*, which encode the proteins polycystin-1 (PC1) or polycystin-2 (PC2), respectively^1,2^. PKD is characterized by the growth of large, fluid-filled cysts from ductal structures in kidneys and other organs, and is among the most common life-threatening monogenic diseases and kidney disorders^3^. Tolvaptan (Jynarque), a vasopressin receptor antagonist that decreases water absorption into the collecting ducts, was recently approved for treatment of PKD in the United States, but only modestly delays cyst growth and has side effects that limit its use^4,5^. At the molecular level, PC1 and PC2 form a receptor-channel complex at the primary cilium that is poorly understood but possibly acts as a flow-sensitive mechanosensor^6–11^. Loss of this complex results in the gradual expansion and dedifferentiation of the tubular epithelium, including increased proliferation and altered transporter expression and localization^12–14^.

As mechanisms of PKD are difficult to decipher in vivo, and murine models do not fully phenocopy or genocopy the human disease, we have developed a human model of PKD in vitro^15–17^. We, together with other groups around the world, have invented methods to derive kidney organoids from human pluripotent stem cells (hPSC), which contain podocyte, proximal tubule, and distal tubule segments in contiguous, nephron-like arrangements^17–20^. Differentiation of these organoids is highly sensitive to the physical properties of the extracellular microenvironment^21^. Organoids derived from gene-edited hPSC with biallelic, truncating mutations in *PKD1* or *PKD2* develop cysts from kidney tubules, reconstituting the pathognomonic hallmark of the disease^15–17^. Interestingly, culture of organoids under suspension conditions dramatically increases the expressivity of the PKD phenotype, revealing a critical role for microenvironment in cystogenesis^16^.

Fluid flow is a major feature of the nephron microenvironment, which is believed to play an important role in PKD^4,5,7,8,22^. However, physiological rates of flow have not yet been achieved in kidney organoid cultures or PKD models. ‘Kidney on a chip’ microphysiological systems provide fit-for-purpose platforms integrating flow with kidney cells to model physiology and disease in a setting that more closely simulates the in vivo condition than monolayer cultures^23–27^. There is currently intense interest in integrating organ on chip systems with organoids, which can be derived from hPSC as a renewable and gene-editable cell source^28–32^. We therefore investigated the effect of flow on PKD in a human organoid on a chip microphysiological system.

## Results

### Flow induces cyst swelling in PKD organoids

**Fig. 1:**
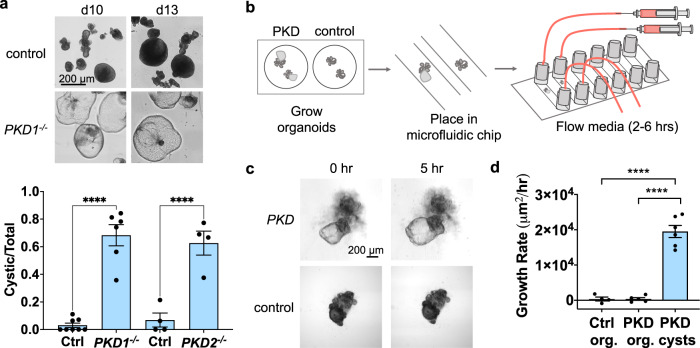
Organoid PKD cysts expand under flow. **a** Representative images of organoids on days following transfer to suspension culture (upper), with quantification (lower) of cyst incidence as a fraction of the total number of organoids (mean ± s.e.m. from *n* ≥ 4 independent experiments per condition; ****p < 0.0001). **b** Schematic of workflow for fluidic condition. **c** Time-lapse phase contrast images of PKD organoids under flow (0.2 dynes/cm^2^), representative of four independent experiments. **d** Average growth rates of control organoids (Ctrl org.), non-cystic compartments of PKD organoids (PKD org.), and cystic compartments of PKD organoids (PKD cysts) under flow (0.2 dynes/cm^2^). Each experiment was performed for 6 h. Cyst growth rate was calculated on an individual basis as the maximal size of the cyst during the time course, divided by the time point at which the cyst reached this size (mean ± s.e.m. from *n* ≥ four independent experiments; each dot represents the average growth rate of organoids in a single experiment. ****p < 0.0001).

Prior to introducing flow, we first confirmed the specificity and timing of the PKD phenotype in static cultures. *PKD1*^*−/−*^ or *PKD2*^*−/−*^ hPSC were differentiated side-by-side with isogenic controls under static, adherent culture conditions to form kidney organoids. On day 18 of differentiation, prior to cyst formation, organoids were carefully detached from the underlying substratum and transferred to suspension cultures in low-attachment plates. Under these conditions, the majority of *PKD1*^*−/−*^ or *PKD2*^*−/−*^ organoids formed cysts within 1–2 weeks, whereas isogenic control organoids rarely formed cysts (Fig. 1a). In repeated trials, the difference between PKD organoids and isogenic controls was quantifiable and highly significant (Fig. 1a). Thus, PKD organoid formed cysts in a genotype-specific manner, strongly suggesting that this phenotype was specific to the disease state. This differs from other types of three-dimensional cultures of epithelial cells, in which hollow ‘cysts’ (spheroids) arise irrespective of PKD genotype and represent a default configuration of the epithelium rather than a disease-specific phenotype^17,33–35^.

To understand how flow affects PKD in organoids, we designed a microfluidic system that allows for live imaging of kidney organoids during the early stages of cyst formation (Fig. 1b). hPSC were first differentiated into organoids under static, adherent culture conditions for 26 days, at which time point tubular structures had formed with small cysts in the PKD cultures. Organoids were then purified by microdissection using a syringe needle^16^, and transferred into gas-permeable, tissue culture-treated polymer flow chambers (0.4 mm height × 3.8 mm width), which were optically clear and large enough to comfortably accommodate organoids and cysts. The channels were pre-coated with a thin layer of Matrigel, and organoids were allowed to attach overnight. PKD and isogenic control organoids were subjected to fluid flow with a wall shear stress of 0.2 dynes/cm^2^, which approximates physiological shear stress within kidney tubules^27,36–38^. In these devices, we observed that cysts in PKD organoids increased in size rapidly under flow (change in area of ~20,000 μm^2^/hr, or ~160 μm/hr in diameter), compared to non-cystic compartments within these organoids, or isogenic control organoids lacking PKD mutations, which did not swell appreciably (Fig. 1c, d and Supplementary Movie 1).

### Diffusion can partially substitute for flow

Having observed that cysts expand under microfluidic conditions, it was important to establish a corresponding static condition lacking flow as a negative control. Initially we utilized the same chambers and syringe pump in the absence of pump activation, which is a commonly used control format for microfluidic experiments. However, we observed that food dye contained within the syringe failed to enter the microfluidic chamber under these conditions (Supplementary Fig. 1a). This indicated a lack of diffusion, which meant that organoids would be exposed only to the volume of media present within the channel of the microfluidic device (~200 µL), which was much lower than the volume they would encounter under fluidic conditions (~60 mL/6 h). Such a static condition could not be readily compared to fluidic conditions to determine the effects of flow, since other parameters such as volume and total solute mass would also be very different.

**Fig. 2:**
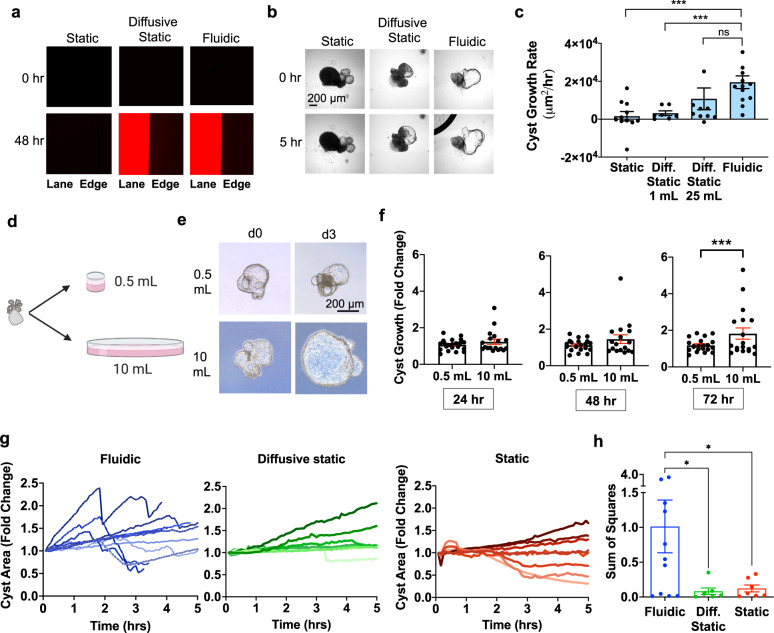
Volume can partially substitute for flow in cyst expansion. **a** Rhodamine dextran (10 kDa) epifluorescence in static (non-diffusive), diffusive static, and fluidic conditions. ‘Lane’ indicates channel interior, and **b** time lapse phase contrast images of cysts in these conditions. Images are representative of *n* ≥ 4 independent experiments. **c** Average growth rates (μm^2^/hr) of cysts in diffusive static condition with different volumes, compared to fluidic or non-diffusive static. Each experiment was performed for 6 h. Cyst growth rate was calculated on an individual basis as the maximal size of the cyst during the time course, divided by the time point at which the cyst reached this size. (*n* ≥ 8 cysts (dots) pooled from two or more independent experiments; ***p < 0.05). **d** Schematic of experiment testing effect of volume vs. pressure on cyst growth. Elements of the image were illustrated using Biorender software under license. **e** Representative phase contrast images and (**f**) quantification of growth rate of cysts suspended in either 0.5 or 10 mL of media under equivalent hydrostatic pressures (mean ± s.e.m. of *n* ≥ 14 cysts per condition pooled from three independent experiments; ***, *p* < 0.05). **g** Growth profiles of individual cysts (lines) over time in microfluidic devices from 0–5 h. Measurements made every 5 min using ImageJ software. Cyst Area was normalized by dividing by the starting area. Data points are from three or more independent experiments. **h** Sum of Squares values from linear regression models were run on each individual cyst (*n* ≥ 7 organoids per condition, pooled from four or more independent experiments; *p* = 0.0342 versus diffusive static and 0.0411 versus static). Error bars, standard error.

To more accurately control for the effects of flow, we designed a diffusive static condition that exposed organoids to an equivalent volume of culture media as in the flow condition. This consisted of a reservoir of media (maximum volume of 25 mL) connected to the microfluidic chip by wider tubing to allow for efficient and uninhibited diffusion of small molecules into the microfluidic channel. In this static format, food dye diffused from the media reservoir into the channel after 2–3 h (Supplementary Fig. 1b). Similarly, rhodamine-labeled dextran (10 kDa) diffused from the media reservoir into the channel and equilibrated with fluidic epifluorescence within 48 h (Fig. 2a).

To further validate this ‘diffusive static’ condition, we varied the volume of media in the reservoir and analyzed cyst growth over a period of 12 h. Cysts exposed to a reservoir containing 1 mL of media expanded at a rate of ~3,000 μm^2^/hr, whereas a reservoir containing 25 mL increased expansion to ~10,000 μm^2^/hr, approximately half the rate observed in the fluidic condition (Fig. 2b, c, Supplementary Movies 2–4). Using the equation *Pressure* = *ρgh*, the hydrostatic pressure on organoids with 1 mL and 25 mL media reservoirs was calculated to be 1174 Pa and 1956 Pa, respectively. As this represented a substantial pressure difference of 5.9 mmHg, we conducted experiments to distinguish between the effects of pressure versus volume on cyst growth. Cystic organoids were suspended in either 500 µl or 10 ml, with a constant fluid column height of 1 cm (Fig. 2d). Cysts exposed to 10 mL of media grew significantly more than those exposed to 500 µL of media (Fig. 2e, f). Thus, media volume was identified as a major determinant of expansion that could partially substitute for flow in this system.

Not all aspects of the fluidic condition were replicated by the diffusive static condition. Time-lapse microscopy under continuous flow revealed that PKD cysts exhibited fluctuating growth profiles, expanding and constricting (deflating) in cyclical, “breath-like” movements. Constrictions occurred rapidly when the cysts appeared to be fully inflated, suggesting that they resulted from rupture of the epithelium, for instance in response to expansive fluid force (Fig. 2g). Growth and constriction events occurred within hours after the initiation of flow, indicating a rapid physical mechanism rather than a slower one based on cell proliferation. This oscillatory behavior was unique to the fluidic condition, and was not observed in either the diffusive static or non-diffusive static conditions, nor in non-cystic controls (Fig. 2g and Supplementary Movie 1). Using the sum of squares method, we found that cyst dynamics (variance in size within an individual structure over time) were much greater in the fluidic condition, compared to either of the static conditions (Fig. 2h). As solute exposure was likely to occur much more rapidly in the fluidic condition, we proceeded to examine solute uptake under these conditions.

### Cysts absorb glucose during flow-mediated expansion

**Fig. 3:**
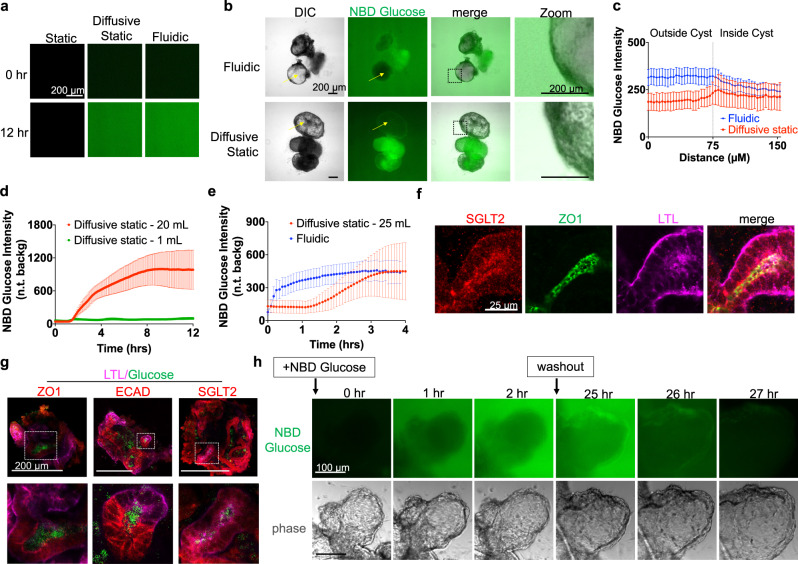
PKD organoids absorb glucose under fluidic and static conditions. **a** NBD Glucose background levels in non-diffusive static, diffusive static, and fluidic conditions after 12 h (representative of three independent experiments). **b** Phase contrast and wide field fluorescence images of organoids in diffusive static and fluidic conditions, 5 h after introduction of NBD glucose (representative of three independent experiments). Arrows are drawn to indicate representative line scans. **c** Line scan analysis of glucose absorption in PKD cysts under static and fluidic conditions after 5 h (mean ± s.e.m. from *n* ≥ 7 cysts per condition pooled from three independent experiments; each n indicates the average of four line scans taken from a single cyst). Background fluorescence levels were calculated at each timepoint by measuring the fluorescence intensity of a square region placed in the non-organoid region of the image. **d** NBD Glucose absorption in the non-cystic compartment of PKD organoid, for Diffusive static 20 mL vs. 1 mL (110 µM NBD Glucose, mean ± s.e.m., *n* ≥ 4 independent experiments), and (**e**) diffusive static 25 mL vs. Fluidic (36.5 µM NBD Glucose, *n* ≥ 5 independent experiments). **f** Confocal fluorescence images of SGLT2 and ZO1 in *PKD1* tubules (representative of three independent experiments). **g** Confocal fluorescent images of NBD Glucose in organoid tubules, fixed and stained with fluorescent cell surface markers (representative of three independent experiments). **h** Time-lapse images of NBD Glucose accumulation in a PKD organoid cyst, followed by washout into media containing unlabeled glucose after 24 h, all performed under continuous flow (representative of three independent experiments).

Kidneys are highly reabsorptive organs, retrieving ~180 L of fluid and solutes per day through the tubular epithelium back into the blood. Glucose is an abundant renal solute and transport cargo, which might explain the effects of media exposure on cyst expansion, but whether kidney organoids absorb glucose is unknown. We therefore studied glucose transport in cysts and organoids using a fluorescent glucose analog, NBD glucose (2-(*N*-(7-Nitrobenz-2-oxa-1,3-diazol-4-yl)Amino)−2-Deoxyglucose). The low height of the channels in our flow devices enabled continuous time lapse imaging of fluorescent molecules without high background fluorescence. Glucose was observed to infiltrate into the devices under both diffusive static as well as fluidic conditions. Epifluorescence of NBD glucose gradually increased and plateaued at similar levels after 12 h in both the diffusive static condition and the fluidic condition, but did not accumulate detectably within the channels in the non-diffusive static condition (Fig. 3a).

When this assay was performed in channels seeded with organoids, PKD cysts absorbed glucose under fluidic and diffusive static conditions (Fig. 3b and Supplementary Movies 5–6). Line scan analysis of these images showed that there was no significant difference in absorption between the fluidic and diffusive static conditions (Fig. 3c). Analysis of glucose absorption in organoid tubules over time confirmed that the volume of media in the static condition was a crucial factor in nutrient absorption (Fig. 3d). Glucose absorption in organoids over time under the diffusive static condition followed an S-shaped absorption curve, whereas glucose levels in the fluidic condition increased rapidly and then plateaued, approximating an exponential curve, but both conditions plateaued at approximately the same maximal level of glucose absorption (Fig. 3e). These studies suggested that flow has no additional effect on glucose absorption in organoids when compared to a static control presenting equivalent total glucose exposure.

Glucose absorption was a general property of kidney organoids. In non-cystic structures, sodium-glucose transporter-2 (SGLT2) was expressed in organoid tubules and enriched at the apical surface, delineated by the tight junction marker ZO-1 (Fig. 3f). Immunofluorescence confirmed that NBD glucose was absorbed into and accumulated inside organoid proximal and distal tubules (Fig. 3g). Immunoblot analysis indicated similar levels of SGLT2 in control and PKD organoid cultures (Supplementary Fig. 2a, b). Cyst-lining epithelia expressed SGLT2, and accumulated glucose both intracellularly as well as inside their lumens (Supplementary Fig. 2c). Intracellular glucose levels were generally higher than extracellular levels, consistent with the tendency of NBD glucose to accumulate inside cells (Supplementary Fig. 3a–c). Although cysts were much less cell-dense than attached non-cystic compartments, cystic and non-cystic compartments accumulated similar total levels of glucose, owing to the larger size of the cysts (Supplementary Fig. 3d). When PKD organoids loaded with NBD glucose were switched into media containing only unlabeled glucose (washout), NBD glucose disappeared rapidly from these structures (Fig. 3h and Supplementary Movie 7–8). Thus, organoids continuously accumulated and released glucose in a dynamic fashion.

### Inhibition of glucose transport blocks cyst growth

**Fig. 4:**
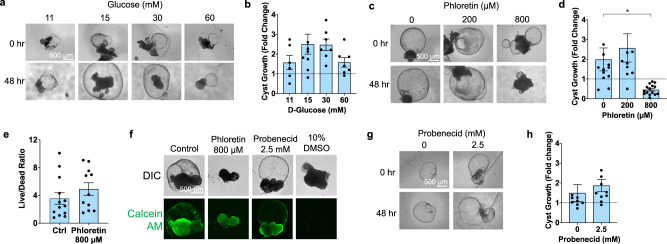
PKD cysts expand in response to glucose stimulation. **a** Representative time lapse brightfield images and (**b**) quantification of change in cyst size in PKD organoids in static suspension cultures containing with D-Glucose concentrations (mean ± s.e.m., *n* ≥ 6 pooled from four independent experiments, each dot indicates a single cyst). **c** Representative time lapse images and (**d**) quantification of PKD organoids in 15 mM D-Glucose treated with phloretin (mean ± s.e.m., *n* ≥ 10 cysts pooled from four independent experiments, *p* = 0.0231). **e** Quantification of maximum intensity projections of live/dead staining in organoids treated with phloretin (mean ± s.e.m., *n* ≥ 11, pooled from two independent experiments, each dot indicates a cystic organoid). **f** Images of live staining with Calcein AM (representative of three independent experiments). **g** Brightfield images and (**h**) quantification of size changes in cystic PKD organoids in 15 mM D-Glucose treated with probenecid (mean ± s.e.m., *n* ≥ 9 pooled from two independent experiments).

In animal models, inhibitors of glucose transport are suggested to have both positive and negative effects in PKD^39,40^. To test functionally whether cyst growth is linked to glucose transport in human organoids, cyst expansion was quantified in increasing concentrations of D-glucose under static conditions (96-well plate). Growth was maximal at 15–30 mM glucose, causing ~50% increase in cyst expansion, relative to lower or higher concentrations (Fig. 4a, b and Supplementary Fig. 4a). Live/dead analysis of cysts treated with 60 mM glucose detected cytotoxicity, explaining the reduction in cyst growth at this higher concentration (Supplementary Fig. 4b–d).

The preceding findings, together with the rapid turnover of glucose in organoids described above, suggested that inhibition of glucose import might enable export mechanisms to dominate, resulting in blockade or even reversal of cyst growth due to osmotic effects. To test this hypothesis, we examined the effects of pharmacological transport inhibitors on cysts in static conditions. Phloretin, a broad spectrum inhibitor of glucose uptake, was tested in 15 mM glucose, and found to decrease cyst size by 77% at a concentration of 800 μM (Fig. 4c, d and Supplementary Fig. 5a). Live-dead staining at 24 and 48 h of phloretin treatment revealed no significant toxicity (Fig. 4d, e and Supplementary Fig. 5b, c). Treatment with either phloridzin, a non-selective inhibitor of both SGLT1 and SGLT2, or with dapagliflozin, a specific inhibitor of SGLT2, reduced cyst growth to baseline at non-toxic doses, further supporting the hypothesis (Supplementary Fig. 5d, e). Net shrinkage of cysts was not observed with phloridzin or dapagliflozin, suggesting either decreased potency of these compounds relative to phloretin, or an off-target effect of phloretin beyond glucose transport that further reduces cyst size. In contrast to SGLT inhibitors, probenecid, an inhibitor of the OAT1 transporter on the basolateral membrane, had no effect on cyst growth compared to controls at non-toxic doses (Fig. 4f–h and Supplementary Fig. 5f). Overall, these findings supported the hypothesis that pharmacological inhibitors of glucose uptake block cyst expansion in the PKD organoid model.

### Organoid cysts polarize outwards

**Fig. 5:**
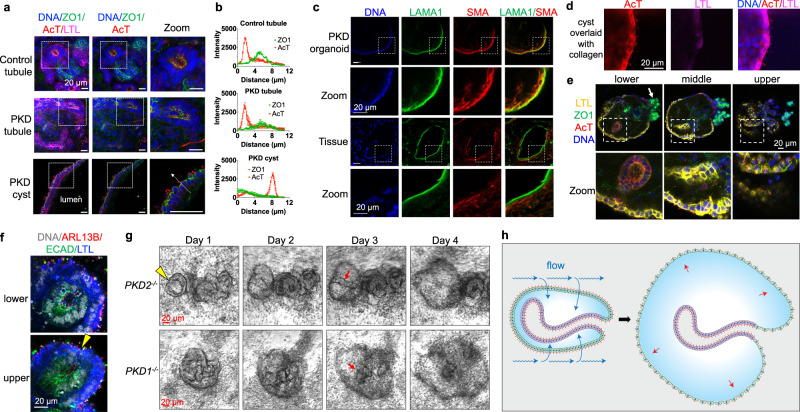
PKD cysts form via expansion of outwards-facing epithelium. **a** Confocal immunofluorescence images of cilia (acetylated α-tubulin, abbreviated AcT) and tight junctions (ZO-1) in proximal tubules (LTL) of PKD and non-PKD organoids, as well as in PKD cyst lining epithelial cells. Dashed arrow indicates how line scans were drawn. Images are representative of three independent experiments. **b** ZO1 and AcT intensity profiles in cysts vs. organoids. Line scans were drawn through cilia from lumen to exterior of structures. (mean ± s.e.m. from *n* = 5 line scans pooled from three organoids or cysts per condition from three independent experiments). **c** Fluorescent images of stromal markers in PKD organoids compared to human kidney tissue from a female patient 50 years of age with autosomal dominant PKD. Scale bars 20 µm. **d** Fluorescent images of cysts after having been overlaid with collagen. Images are representative of three independent experiments. **e** Z-stack confocal images of early (day 30) PKD organoid cyst in adherent culture. Zoom shows boxed region. White arrow indicates a podocyte cluster continuous with the peripheral epithelium. Images are representative of three independent experiments. **f** Close-up image showing peripheral epithelium of control (non-PKD) organoid in adherent culture. Yellow arrowhead indicates region of epithelial invagination. Images are representative of three independent experiments. **g** Phase contrast time-lapse images showing formation of PKD cysts from non-cystic structures in adherent cultures. Red arrows indicates tubular structures internal to the peripheral cyst. Images are representative of three independent experiments. **h** Schematic model of absorptive cyst expansion in organoids. Fluid flow (blue arrows) is absorbed into outwards-facing proximal tubular epithelium, which generates internal pressure that drives expansion and stretching of the epithelium (red arrows). A simplified organoid lacking podocytes or multiple nephron branches is shown for clarity.

Some previous studies have suggested that cyst expansion may be due to increased secretory (basolateral-to-apical) solute transport^41–44^. However, glucose transport in the proximal tubule is predominantly reabsorptive (apical-to-basolateral) rather than secretory. To better understand the directionality of transport within organoids, we determined the apicobasal polarity of tubules and cysts using antibodies against tight junctions and cilia. In both PKD and control organoids, the ciliated surface of these tubules faced inwards (Fig. 5a). Surprisingly, however, PKD cysts were polarized with the apical ciliated surface facing outwards towards the media and exposed to flow (Fig. 5a). Thus, the external cyst surface resembled the apical surface of a tubule in this system. Line scan analysis confirmed this inverted polarization, with primary cilia and tight junction intensity profiles reversed in organoids vs. cysts (Fig. 5b).

Close examination of PKD organoid cysts revealed that a subpopulation of these contained a layer of cells expressing alpha smooth muscle actin immediately beneath the cyst-lining epithelium, which formed a laminin-rich basement membrane (Fig. 5c). In contrast, in human kidney tissue the basement membrane and myofibroblast-like cells surrounded cysts externally (Fig. 5c). Thus, apical cell polarity aligned opposite the basement membrane in both systems. Simple spheroids of Madine-Darby Canine Kidney cells in suspension culture polarize outwards, but can reverse apicobasal polarity from outwards to inwards when embedded in collagen^34^. When PKD cysts in organoids were overlaid with collagen, however, cyst polarity remained inverted and did not repolarize with the ciliated surface facing away from the extracellular matrix, indicating that organoid cyst polarity was deeply entrenched and governed by more dominant, internal cues (Fig. 5d).

The observation that cysts polarized outwards seemed counter-intuitive, as tubule structures in human kidney organoids typically polarize inwards, with tight junctions and apical markers abutting one another from diametrically opposed epithelia (as shown in Fig. 5a)^17^. To resolve this conundrum, we closely examined PKD organoids in three-dimensional confocal image z-stacks. *Lotus tetragonolobus* lectin (LTL), which is expressed more strongly in tubules than in cysts, was used to label the epithelium, while primary cilia and ZO-1 were used to indicate cell polarity. These experiments revealed that young cysts comprised epithelial spheroid structures (predominantly LTL^+^) with underlying tubular infolds, which faced inwards (Fig. 5e). We further examined organoids without cysts (controls) in confocal microscopy z-stacks. We noted that epithelium lining the periphery of these organoids faced outwards, whereas ‘tubules’ internal to organoids were invaginations of this peripheral epithelium (Fig. 5f and Supplementary Fig. 6a, b). The innermost regions of these invaginated tubules were enriched for ECAD, a marker of distal tubule, whereas the external peripheral epithelia were enriched for LTL, a marker of proximal tubule (Fig. 5f and Supplementary Fig. 6a). Thus, organoids constituted a continuous, proximal-to-distal epithelium, with the apical surface polarized outwards on the peripheral (more proximal) epithelium and inwards in the internal (more distal) epithelium of the structure.

To observe the process of cyst formation in real time, we collected time-lapse images of young PKD organoids undergoing cystogenesis over eight days in culture. Consistently, cysts formed at the periphery of the organoids (Fig. 5g, Supplementary Fig. 7a, b, and Supplementary Movie 9). During the early stages of cystogenesis, tubular structures remained visible inside the cysts as they expanded (Fig. 5g, Supplementary Fig. 7a, b, and Supplementary Movie 9). Thus, time-lapse imaging supported the idea that cysts formed from the peripheral epithelium of the organoids that faced outwards towards the media, rather than from the internal tubular invaginations, which tended to stay anchored (Fig. 5h). This was consistent with an absorptive mechanism mediated by the peripheral epithelium.

### Absorptive cysts form in vivo

**Fig. 6:**
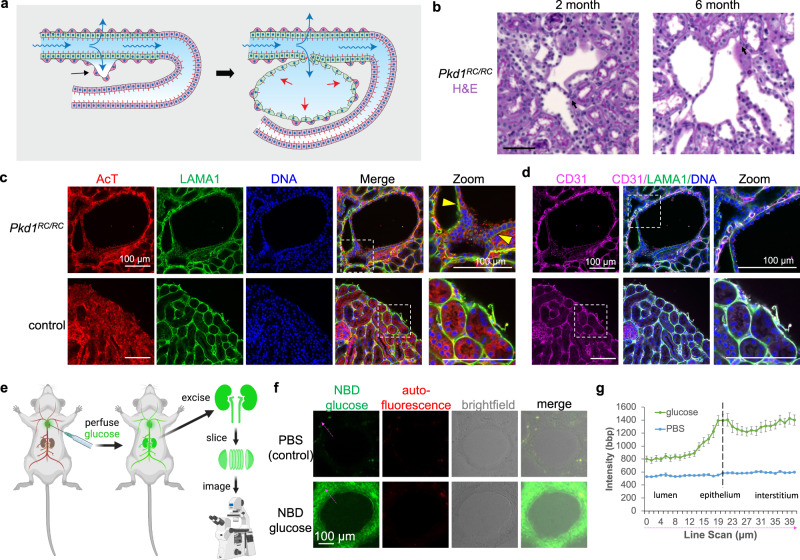
PKD cysts in vivo absorb glucose into the surrounding interstitium. **a** Hypothetical schematic of absorptive cyst formation in kidney tissue. Fluid (blue arrows) is absorbed through proximal tubules into the underlying interstitium, which partially detach from the epithelium. The tubules then expand and deform to fill the interstitial space, reaching a low-energy conformation in which the withheld volume is ultimately transferred back into the luminal space of the nascent microcyst. A simplified model is shown and represents one possible explanation of the findings. **b** PAS stains of 2-month-old and 6-month-old *Pkd1*^*RC/RC*^ mice (C57BL/6 J background). Scale bars 50 µm. Images are representative of 4 animals per condition (two male and two female). **c** Confocal images of stromal basement membrane (LAMA1) with cilia (AcT) or (**d**) endothelial cells (CD31) in *Pkd1*^*RC/RC*^ versus control (*Pkd1*^*+/+*^) 2-month-old mice. All mice were C57BL/6 J background. Yellow arrowheads indicate areas of detached or expanded interstitium surrounding the cyst. Images are representative of four animals per condition (two male and two female). **e** Schematic of glucose uptake assay, illustrated using Biorender software under license. **f** Representative images and (**g**) line scan analysis of PKD cysts after perfusion with fluorescent NBD glucose or unlabeled PBS control (mean ± s.e.m., *n* ≥ 17 cysts per condition pooled from a total of three female and two male *Pkd1*^*RC/RC*^ mice of C57BL/6J background). Dashed magenta arrows indicate how line scans were drawn.

It is important to understand how these findings in organoids might relate to PKD cyst formation in vivo, where cyst-lining epithelia face inwards rather than outwards. Microcysts smaller than 1 mm diameter and undetectable by magnetic resonance imaging are numerous in kidney sections from patients with early stages of PKD, and are proposed to form as focal outpouchings of tubular epithelium^45,46^. If such an outpouching remained connected to a small segment of the original tubule via apical junctions, it could accumulate fluid through tubular reabsorption. The preceding suggested a possible model for cyst formation in vivo (Fig. 6a). Absorption of glucose through the apical surface of the tubular epithelium is followed by water along the osmotic gradient via paracellular or transcellular routes to maintain balanced concentrations on either side of the epithelium. There is a lack of appropriate outlet for this absorptive activity, creating a pressure within the interstitium and leading to its detachment from neighboring tubules, which undergo deformation and expansion to fill the resultant interstitial space. This process continues as the cyst grows, and may be exacerbated by the gradual loss or detachment of associated peritubular capillaries (which reduces the absorptive sink), and by growth of interstitial mesenchymal stromal cells, which provide a scaffold and synthesize extracellular matrix to accommodate the expanding epithelium.

To investigate the plausibility of such a mechanism in vivo, we analyzed microcysts in the *Pkd1*^*RC/RC*^ mouse strain, which has a hypomorphic *Pkd1* gene mutation orthologous to patient disease variant *PKD1* p. R3277C, and manifest a slowly progressive PKD during adulthood over a period of several months^47,48^. Histology sections and confocal images of 2-month-old mouse tissue revealed continuous basement membranes between tubules and microcysts, consistent with the possibility that microcysts form from tubular outpouchings that remain capable of absorption through the wall of the neighboring tubule (Fig. 6b, c). While much of these microcysts remained tightly associated with peritubular capillaries, suggesting that they continue to reabsorb, portions of the epithelium appeared to have detached from the endothelium, resulting in areas of fluid accumulation or interstitial expansion (Fig. 6b–d).

To determine whether PKD cysts absorbed glucose in vivo, we devised a methodology to inject mice with NBD glucose and immediately retrieve their kidneys (Fig. 6e). Fluorescence microscopy analysis of kidney tissue sections revealed that cyst-lining epithelia and the surrounding interstitium readily took up NBD glucose (Fig. 6f–g). Thus, cysts remained absorptive in vivo and PKD kidneys as a whole readily accumulated glucose.

## Discussion

Coupling the structural and functional characteristics of organoids with the controlled, microfluidic microenvironments of organ-on-a-chip devices is a promising approach to in vitro disease modeling^28^. Our study combines CRISPR-Cas9 gene editing to reconstitute disease phenotype with organoid-on-a-chip technology to understand the effect of flow, which is difficult to assess in vivo (where it is constant) and has hitherto been absent from kidney organoid models at physiological strength. The ‘human kidney organoid on a chip’ microphysiological system described here incorporates organoids with PKD mutations in a wide-channel format, which allows liquid to flow over the organoids, similar in geometry to other recently described organoid flow systems^29,30^. At the core of this system are human organoids that strikingly recapitulate the genotype-phenotype correlation in PKD. This is fundamentally different from other other types of generic spheroids that form in vitro as a default configuration of the epithelium. While certain aspects of the organoid system differ from in vivo, we do not see a plausible explanation wherein the genotype-phenotype correlation is preserved, but the entire system is somehow irrelevant or opposite to the fundamental mechanism of PKD. Rather, the system is teaching us which aspects of PKD are most important for the phenotype. The system can be readily assembled from commercially available components, and produces a shear stress associated with the physiological range found in human kidney kidney tubules^27,36–38^. This is ~6-fold greater than the maximum rate of 0.035 dyn/cm^2^ used in a previous kidney organoid-on-a-chip device, a shear stress that was nevertheless sufficient to stimulate expansion of vasculature within the device when compared to static conditions^29^, and to induce dilation of tubular structures derived from hPSC with mutations associated with autosomal recessive PKD (ARPKD)^49^. The physiological relevance of such low flow rates is not clear, and the cohort of ARPKD cell lines that was studied includes hPSC previously generated by our laboratory that we found to lack definitive ARPKD mutations^50^. It is nevertheless interesting and encouraging that flow over the organoids was capable of inducing swelling in both systems.

Importantly, we have also developed a static module using the same basic chip that is capable of natural diffusion from a syringe reservoir. This enables us to distinguish the effects of flow from those of exposure to fluid volume and mass of reabsorbable solute, which is difficult to achieve in conventional systems with limited diffusion such as tightly connecting a reservoir to a Luer lock syringe. Our discovery that volume can partially substitute for flow is reminiscent of a recent study in which immersion in >100-fold volumes induced three-dimensional morphogenesis of intestinal epithelial cells similar to flow^51^. In contrast, increased volume was unable to substitute for flow in the aforementioned study of endothelial expansion in kidney organoid cultures. This may reflect a sensitivity of vascular cells to fluid shear stress, or alternatively the limited volumes possible in closed loop systems^29^. In addition to volume, hydrostatic pressure is increased in our diffusive static condition, which may play a role in PKD phenotype^52^. Of note, cysts in our diffusive static condition did not exhibit the dramatic oscillations in size observed under flow, indicating roles for flow-induced mechanoregulation that cannot be readily replicated by diffusion effects, for instance involving stretch-activated ion channels.

Our findings indicate that flow, volume, and solute concentrations are positive regulators of cyst expansion. Cystogenesis can be enhanced through mechanisms of tubular absorption and glucose transport. A limitation of these systems is that the perfusion passes over the organoids, rather than through them as it does through tubules in vivo. However, as peripheral epithelia in our organoids face outwards towards the media, the net result is for the apical surface to be in contact with the directional flow, similar to the epithelium of a tubule in vivo. This fortuitously enables us to assess reabsorptive function, the primary characteristic of kidney tubular network, which fluxes ~180 L through its apical surface every day. In this regard, the arrangement in the organoid system may have greater functional relevance than spheroid systems in which cyst polarity faces inwards but the liquid is trapped inside with no possibility of perfusion (unlike the arrangement in the kidneys).

The observation that PKD cysts can form inside-out, such that the secretion (basolateral-to-apical transport) would occur in the opposite direction from cysts in vivo, argues against secretion as the critical driver of cystogenesis in this system^43^. Our experiments in animals also demonstrate that kidney cysts remain reabsorptive even in advanced PKD. In our studies in vivo, we also made the interesting discovery that the tubular epithelium detaches focally from the underlying interstitium during pre-cystic stages of disease, which may reflect the consequences of a possible absorptive phenotype. Studies of PKD in living animals, however, carry significant constraints for studying mechanism. Kidneys are concealed within the body, preventing detailed time-lapse microscopy, and perturbing renal absorption is experimentally challenging and causes complex side effects. Demonstrating glucose absorption in cystic kidneys in vivo, and showing interstitial detachment, as we have done, required significant methods development and careful analysis. Further methods development and more detailed studies are required to causally link absorption, interstitial detachment, and cyst formation in vivo. Nevertheless, it is clear that renal cysts can continue to absorb glucose, even in vivo, and in organoids, glucose absorption is linked to the PKD phenotype, which is demonstrably specific to the genotype and thus mechanistically relevant.

These findings are consistent with macropuncture studies showing that wall pressures inside PKD cysts in vivo resemble their originating nephron segments, and studies of excised cysts in vitro, which demonstrate that the epithelium is slowly expanding and absorptive under steady-state conditions^44,45^. In a more recent clinical analysis, patients with ADPKD demonstrated lower excretion of renally secreted solutes, rather than higher levels of secretion^53^. Drugs that activate CFTR, which is hypothesized to drive a secretory phenotype in PKD, have shown promise in treating PKD in mice, rather than exacerbating the disease, which is also inconsistent with a secretory hypothesis^54^. Indeed, a phenotype related to absorption is a much more natural fit for the specialized properties of kidney epithelia (which are predominantly absorptive) than secretion. This is not to say that secretion cannot be a causative mechanism in PKD cystogenesis, but rather that absorption can also play a critical role. In our model, absorption of fluid into the interstitium creates space for epithelia to expand and fill. During this process of expansion and space filling by the epithelium, which is triggered by changes within the microenvironment surrounding the tubules, it is conceivable that secretory processes play a role.

Previously, we observed that transfer of PKD organoids from adherent cultures into suspension cultures was associated with dramatically increased rates of cystogenesis^16^. Our current findings add greatly to our understanding of this phenomenon. Upon release from the underlying substratum, the peripheral organoid epithelium grows out and envelops the rest of the organoid^16^. This forms an enclosed, outwards-facing structure in an ideal conformation to absorb fluid from the surrounding media and expand into a cyst. Although we did not detect differences in the levels of SGLT2, differences may exist in SGLT2 activity, or in the levels or activity of other transporters involved in absorption, resulting in increased absorptive flux in PKD epithelia, compared to non-PKD. Alternatively, there might exist a difference in the pliability of PKD epithelia versus non-PKD epithelia undergoing equivalent levels of absorptive flux. We note that polycystin-2 is a non-selective cation channel expressed at the apical plasma membrane^9,10^, which could conceivably play a role in transporter function and reabsorption. The polycystin complex may also possess force- or pressure-sensitive mechanoreceptor properties, which could regulate the epithelial response to fluid influx^4,5,7,8,22,52^.

Although we favor a direct role for glucose absorption in driving cyst expansion, glucose transport could also function separately of water transport to impact cyst formation, for instance by altering mitochondrial metabolism or signaling changes to the actin cytoskeleton, which could promote cystogenesis regardless of which direction the cells face^55–58^. Of note, cysts form not only in the proximal tubules that are primarily responsible for glucose reabsorption, but also in the collecting ducts, where they can reach very large sizes. As cysts can and must originate from these very different epithelial cell types, the process of cystogenesis is not likely to be explained by a simple absorption/secretion ratio for any one solute. One goal for future development of our PKD organoid system is to incorporate collecting ducts, as this lineage is important to PKD cystogenesis but does not mature in human kidney organoid cultures^17,59,60^.

A limitation of the current system is that the organoid phenotype is limited to biallelic mutants, in which disease processes are greatly accelerated^61–63^. In contrast, germline mutations in PKD patients are monoallelic, and phenotypes take decades to develop, likely due to the necessity of developing ‘second hit’ somatic mutations in the second allele^64,65^. The current system involving biallelic mutants may more closely phenocopy early-onset autosomal recessive PKD than late-onset autosomal dominant PKD, which should be considered when extrapolating these findings into a clinical context^16^. Generation of well-controlled allelic series of PKD organoids, together with methodologies to model the acquisition of somatic mutations, may ultimately produce human organoid models with greater fidelity to autosomal dominant PKD.

Canagliflozin (Invokana), an inhibitor of SGLT2, has recently been approved for the treatment of type 2 diabetes, and appears to have a protective effect in the kidneys^66,67^. SGLT inhibitors have not yet been tried in patients with PKD. Our findings suggest that blocking SGLT activity could reduce proximal tubule cysts by preventing glucose reabsorption. However, this would also expose the collecting ducts downstream to higher glucose concentrations. Indeed, it was previously suggested that inhibition of glucose transport reduces PKD in the Han:SPRD rat because its cysts originate from proximal tubules, whereas the same treatments in the PCK rat worsen PKD because its cysts originate in more distal nephron segments^39,40^. Caution must therefore be exercised when considering how to conduct human clinical trials for PKD with SGLT inhibitors.

In summary, we have developed a microfluidic kidney organoid module that enables detailed studies of renal tubular absorption and PKD cyst growth. The cyst-lining epithelium in this system is exposed to flow in a mirror image of the nephron structure in vivo. Using this system, we have identified glucose levels and its transport into cyst structures as a driver of cystic expansion in proximal nephron-like structures. Therapeutics that modulate reabsorption may therefore be beneficial in reducing cyst growth in specific nephron segments, with relevance for future PKD clinical trials^4,66^.

## Methods

### Ethics

Research complied with all relevant ethical regulations. Human PKD kidney tissue (nephrectomy) was obtained with informed consent under a human subjects protocol approved by the University of Washington Institutional Review Board. No compensation was provided to study participants.

### Kidney organoid differentiation

Work with hPSC was performed under the approval and auspices of the University of Washington Embryonic Stem Cell Research Oversight Committee. Specific cell lines used in this study are described below and are sourced from commercially available hPSC obtained with informed consent. hPSC stocks were maintained in mTeSR1 media with daily media changes and weekly passaging using Accutase or ReLeSR (STEMCELL Technologies, Vancouver). 5,000–20,000 hPSCs per well were placed in each 24-well plate pre-coated with 300 µL of DMEM-F12 containing 0.2 mg/mL Matrigel and sandwiched the following day with 0.2 mg/mL Matrigel in mTeSR1 (STEMCELL Technologies, Vancouver) to produce scattered, isolated spheroid colonies. 48 hrs after sandwiching, hPSC spheroids were treated with 12μM CHIR99021 (Tocris Bioscience) for 36 h, then changed to RB (Advanced RPMI + 1X Glutamax + 1X B27 Supplement, all from Thermo Fisher Scientific) after 48 h, and replaced with fresh RB every 3 days thereafter.

### Organoid perfusion in microfluidic chip

Ibidi μ-Slide VI^0.4^ were coated with 3.0% Reduced Growth Factor Geltrex (Life Technologies) and left at 37 °C overnight to solidify. Kidney organoids (21–40d) were picked from adhered culture plates, pipetted into the slide channels (2–3 per channel) with RB, and left for 24 hrs at 37 °C to attach. Organoids were distributed randomly within the channel. For the fluidic condition, 60 mL syringes filled with RB were attached to channels using clear tubing (Cole-Parmer, 0.02'' ID, 0.083'' OD). A clamp was used to close off the tubing, and the media in the syringe was changed to 25 mL RB + 36.5 μM 2-NBD-Glucose fluorescent glucose (Abcam ab146200). A Harvard Apparatus syringe infusion pump was used to direct media flow into microfluidic chip at 160 μL/min (0.2 dynes/cm^2^). Media was collected at the outlet and filtered for repeated use. For the static condition, a 25 mL syringe containing RB was attached to the channel using wide clear tubing (Cole-Parmer, 0.125'' ID, 0.188'' OD). The syringe was detached momentarily, the plunger removed, and the open syringe reattached and filled slowly with 25 mL RB + 36.5 μM 2-NBD-Glucose. From this point on, diffusion of the fluorescent glucose began from the open syringe into the channel via the tubing. Alternatively, NDB-glucose was substituted with food dye (invert sugar, 360 g/mol), or alternatively the organoids were perfused with media in the absence of any additives.

### Image/video collection

Image collection was performed on a Nikon Ti Live-Cell Inverted Widefield microscope inside of an incubated live imaging chamber supplemented with 5% carbon dioxide. Experiments in microfluidic devices were recorded for 6 h. During this time, cysts changed in volume (grew and shrank) and in some cases were destroyed due to bubbles arising in the tubing. Cyst growth rate in microfluidic devices was therefore calculated on an individual basis, when each cyst reached its maximal volume, which varied for each sample from 1 h to 5 h after the start of the experiment. For longer-term experiments conducted in static 96-well cultures, organoids were imaged at regular intervals (typically 24 h) and analyzed at the endpoint indicated in the figure graphs. Phase contrast and GFP (200 ms exposure) images were taken every 5 min for a maximum of 12 h. Images of fixed samples were collected on a Nikon A1R point scanning confocal microscope.

### Animal studies

Kidney tissue from *Pkd1*^RC/RC^ mice maintained in C57BL/6 J background (gift of Mayo Clinic Translational PKD Center) and C57BL/6 J controls were utilized. In order to investigate the process of cystogenesis, younger *Pkd1*^RC/RC^ mice 6–7 weeks of age, along with wild-type C57BL/6 J mice of the same age were used. Kidneys were harvested after systemic perfusion with ice-cold PBS, followed by fixation with paraformaldehyde fixative and immersion in 18–30% sucrose at 4 °C overnight. Tissues were embedded and frozen in optimal cutting temperature compound (OCT, Sakura Finetek, Torrance, CA). Cryostat-cut mouse kidney sections (5–10 μm) were stained for acetylated α-tubulin, laminin-1, and CD31 (see “Immunostaining” for primary antibodies and dilutions).

For perfusion experiments, NBD Glucose was freshly dissolved in PBS to a concentration of 1 mM. Freshly sacrificed *Pkd1*^*RC/RC*^ mice (>8 months old) were incised through the chest and nicked at the vena cava with a 27-gauge needle. Keeping pressure on the vena cava, mice were perfused systemically through the heart with a syringe containing 10 ml of PBS, followed by a second syringe containing 5 ml of either PBS alone (control) or PBS + 1 mM NBD-Glucose. Kidneys were harvested immediately and embedded fresh without fixation or sucrose equilibration in OCT. Cryostat-cut mouse kidney sections (20 μm) were mounted in OCT and imaged on a confocal microscope with 10X objective. All animal studies were conducted in accordance with all relevant ethical regulations under protocols approved by the Institutional Animal Care and Use Committee at the University of Washington in Seattle. Mice were maintained on a standard diet under standard pathogen-free housing conditions, with food and water freely available.

### Immunostaining

Immunostaining followed by confocal microscopy was used to localize various proteins and transporters in the cysts and organoids. Prior to staining, an equal volume of 8% paraformaldehyde was added to the culture media (4% final concentration) for 15 mins at room temperature. After fixing, samples were washed in PBS, blocked in 5% donkey serum (Millipore)/0.3% Triton-X-100/PBS, incubated overnight in 1% bovine serum albumin/0.3% Triton-X-100/10μM CaCl_2_/PBS with primary antibodies, washed, incubated with Alexa-Fluor secondary antibodies (Invitrogen), washed and imaged. Primary antibodies or labels include acetylated α-tubulin (Sigma T7451, 1:5000), ZO-1 (Invitrogen 61-7300, 1:200), Biotinylated LTL (Vector Labs B-1325, 1:500), E-Cadherin (Abcam ab11512, 1:500), SGLT2 (Abcam ab37296, 1:100), laminin-1 (Sigma L9393, 1:50), alpha smooth muscle actin (Sigma A2547, 1:500), CD31 (BD Biosciences 557355, 1:300). Fluorescence images were captured using a Nikon A1R inverted confocal microscope with objectives ranging from 10X to 60X.

### Statistical analysis

Experiments were performed using a cohort of PKD hPSC, previously generated and characterized, including three *PKD2*^*−/−*^ hPSC lines and three isogenic control lines that were subjected to CRISPR mutagenesis (gRNA CGTGGAGCCGCGATAACCC) but were found to be unmodified at the targeted locus by Sanger sequencing of each allele and immunoblot^16,17^. Altogether these represented two distinct genetic backgrounds, genders, and cell types: (i) male WTC11 iPS cells (Coriell Institute Biobank, GM25256, two isogenic pairs) and (ii) female H9 ES cells (WiCell, Madison Wisconsin, WA09, one isogenic pair). Quantification was performed on data obtained from experiments performed on controls and treatment conditions side by side on at least three different occasions or cell lines (biological replicates). Error bars are mean ± standard error (s.e.m.). Statistical analyses were performed using GraphPad Prism Software. To test significance, *p*-values were calculated using two-tailed, unpaired or paired *t*-test (as appropriate to the experiment) with Welch’s correction (unequal variances). For multiple comparisons, standard ANOVA was used. Statistical significance was defined as *p* < 0.05. Exact or approximate *p-*values are provided in the figure legends in experiments that showed statistical significance. For traces of cysts over time, the least squares progression model was applied to fit the data to lines in GraphPad Prism. Line scans of equal length were averaged from multiple images and structures based on raw data intensity values in the GFP channel. Lines were drawn transecting representative regions of each structure (e.g. avoiding heterogeneities, brightness artifacts, or areas where cysts and organoids overlapped), placed such that the first half of each line represented the background in the image. The intensity of each point (pixel) along the line was then averaged for all of the lines, producing an averaged line scan with error measurements. Arrows are provided in representative images showing the direction and length of the line scans used to quantify the data. Unless otherwise noted, raw intensity values (bytes per pixel) were were used without background subtraction.

### Hydrostatic pressure calculation

The following calculation was performed:

$${{{{{\rm{Pressure}}}}}}=\rho {{{{{\rm{gh}}}}}}=\left(997\frac{{kg}}{{m}^{3}}\right)\left(9.81\frac{m}{{s}^{2}}\right)\left({{{{\boldsymbol{{{{{\mathscr{x}}}}}}}}}}\,m\right)={Pressure}\left(\frac{{kg}}{m\cdot {s}^{2}}\right)$$

The height from channel to top of media in reservoir was measured to be:

Static 1 mL: ~12 cm

Static 25 mL: ~20 cm

Therefore, the calculation for each of these conditions was:

$$Pressur{e}_{1mL}	=\rho {{{{{\rm{gh}}}}}}=\left(997\frac{kg}{{m}^{3}}\right)\left(9.81\frac{m}{{s}^{2}}\right)(0.12\;m) \\ 	=1173.7\left(\frac{kg}{m\cdot {s}^{2}}\right)\left(\frac{mmHg}{133.32\,Pa}\right)=8.8\,mmHg$$

$$Pressur{e}_{25mL}	=\rho {{{{{\rm{gh}}}}}}=\left(997\frac{kg}{{m}^{3}}\right)\left(9.81\frac{m}{{s}^{2}}\right)(0.20\,m) \\ 	=1956.1\left(\frac{kg}{m\cdot {s}^{2}}\right)\left(\frac{mmHg}{133.32\,Pa}\right)=14.7\,mmHg$$

This amounted to a total difference in pressure of (14.7–8.8 = 5.9) mmHg.

### Reporting summary

Further information on research design is available in the Nature Portfolio Reporting Summary linked to this article.

## Supplementary information

Supplementary Information

Description of Additional Supplementary Files

Movie 1

Movie 2

Movie 3

Movie 4

Movie 5

Movie 6

Movie 7

Movie 8

Movie 9

Reporting Summary

## Data Availability

The main data supporting the results in this study are available within the paper and its supplementary information. The raw and analysed datasets generated during the study are too large and complex to be publicly shared (numerous cell lines, replicates, images, blots, and experiments, maintained and analysed in specialized file formats and with unique identifiers). Datapoints are shown as dots in the plots provided in this paper and the Supplement. All datasets, including raw data and statistical analysis, are available upon reasonable request from the corresponding author. PKD mutant cell lines used in this study may be obtained from the corresponding author upon request and in accordance with material transfer agreements from the University of Washington and any third-party originating sources. Source data are provided with this paper.

## Source data

Source Data
