# Supplementary material for: Glucose absorption drives cystogenesis in a human organoid-on-chip model of polycystic kidney disease
Source: Nat Commun. 2022 Dec 23;13:7918. doi: 10.1038/s41467-022-35537-2 (PMC9789147; doi:10.1038/s41467-022-35537-2)
Supplement: Supplementary file 1 — Supplementary Information [file 41467_2022_35537_MOESM1_ESM.pdf]

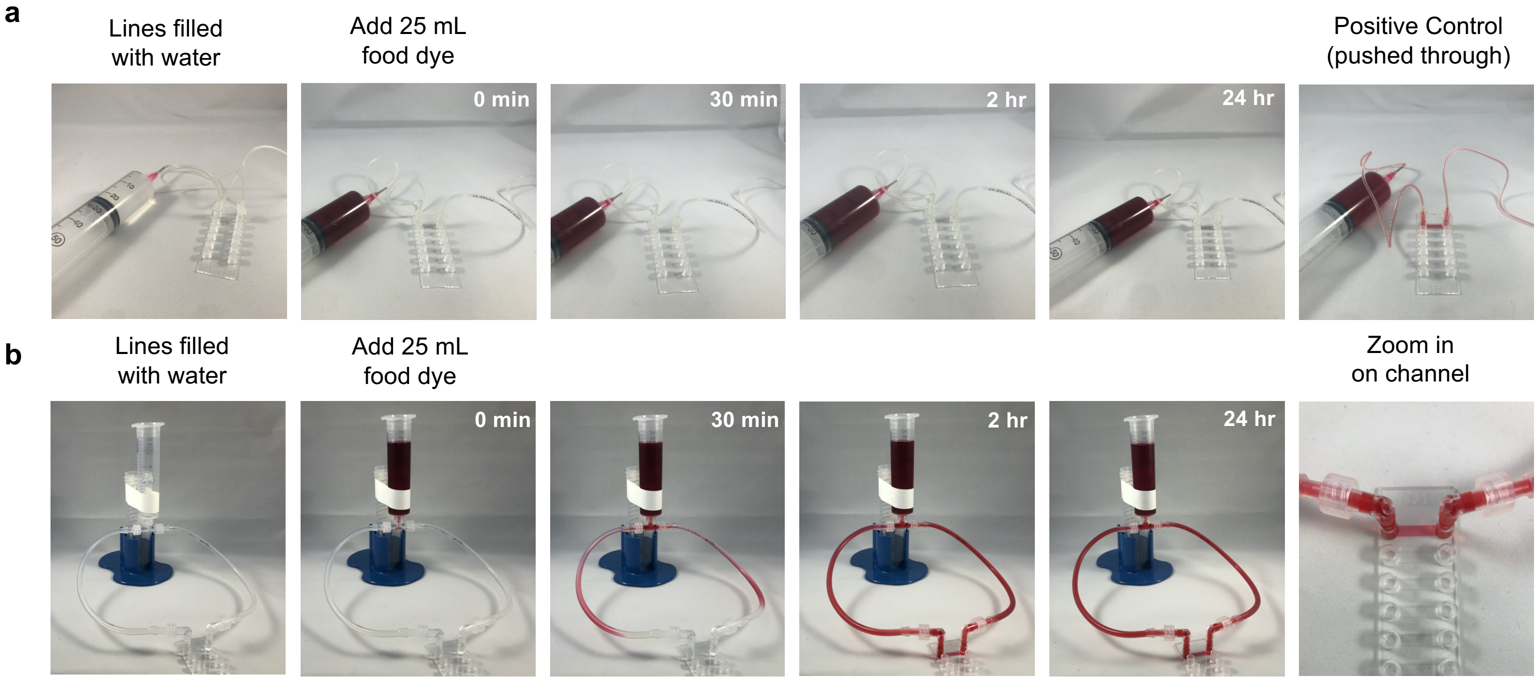

Supplementary Figure 1. A static condition that allows diffusion promotes cyst expansion. (a) Photographs of food dye moving through 'non-diffusive' static condition incorporating a luer lock syringe and **(b)** 'diffusive' static condition incorporating media reservoir suspended over microfluidic chip and connected by wide tubing.

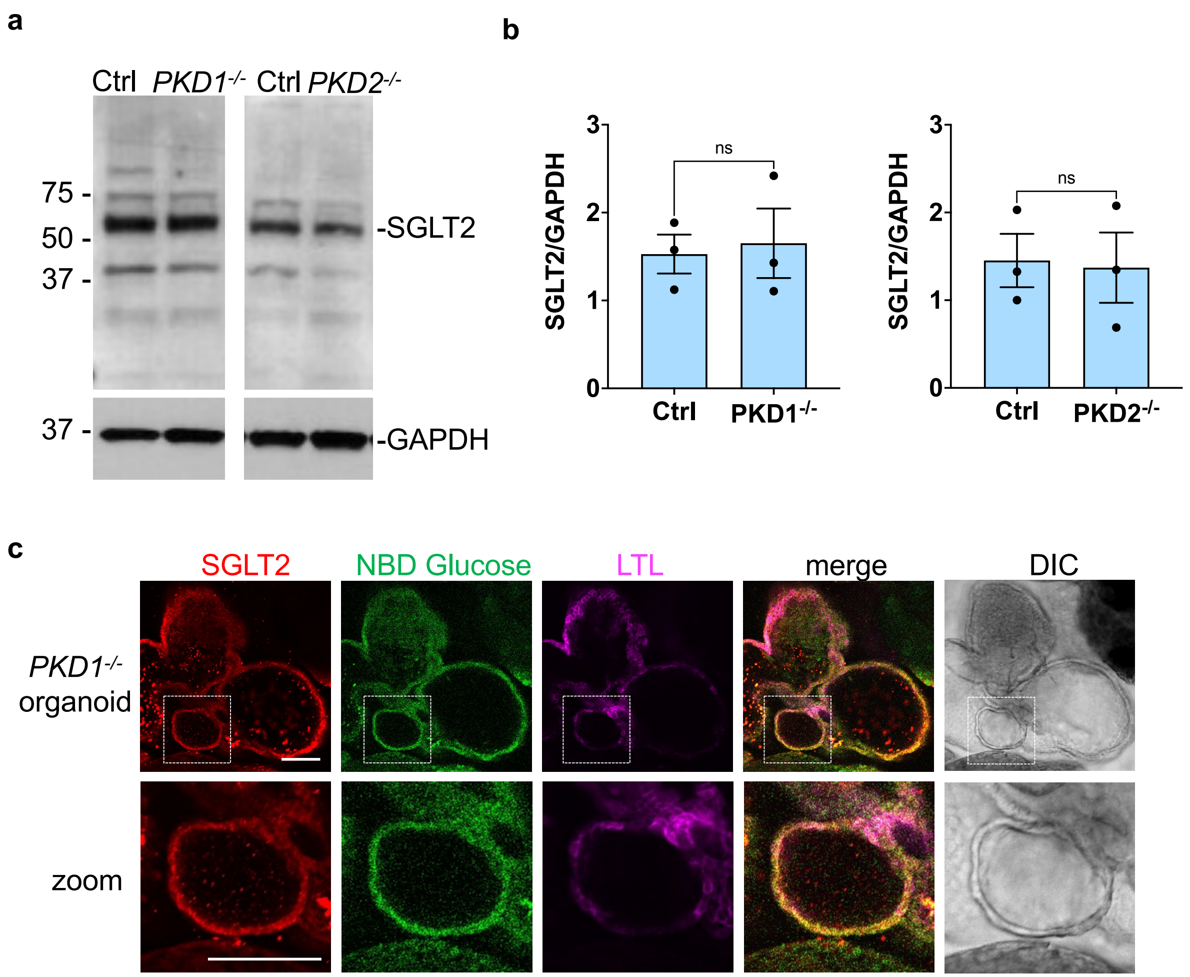

Supplementary Figure 2. SGLT2 is expressed in PKD cysts and organoids. (a) Representative immunoblots and **(b)** quantification of SGLT2 levels in organoid cultures (mean ± s.e.m. from three independent experiments). **(c)** Representative confocal images of SGLT2 and NBD Glucose in PKD cysts, with zoom of boxed white region. Scale bars 100 µm.

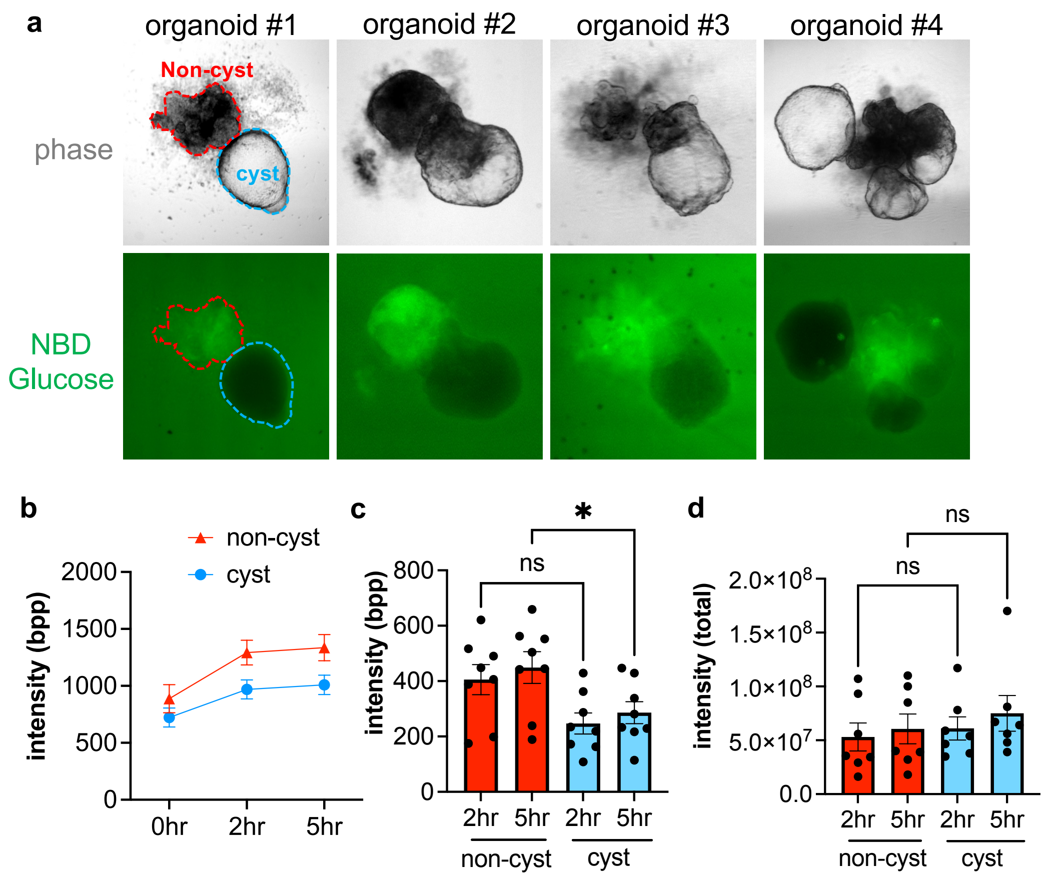

Supplementary Figure 3. Glucose accumulates in organoids and cysts. (a) Representative confocal images of organoids following 5hr of exposure to NBD Glucose. Red and blue dotted lines mark demonstrate tracing of non-cyst compartment and cyst, respectively. **(b)** Raw and **(c)** background subtracted NBD glucose fluorescence intensity over time in non-cyst compartments and cysts (mean ± s.e.m. from seven organoids, pooled from three independent experiments; *, $p < 0.05$). **(d)** Total glucose levels in these structures, calculated as (Area * Mean Intensity), with background subtraction based on 0 hr (mean ± s.e.m. from seven organoids, pooled from three independent experiments; *, $p < 0.05$).

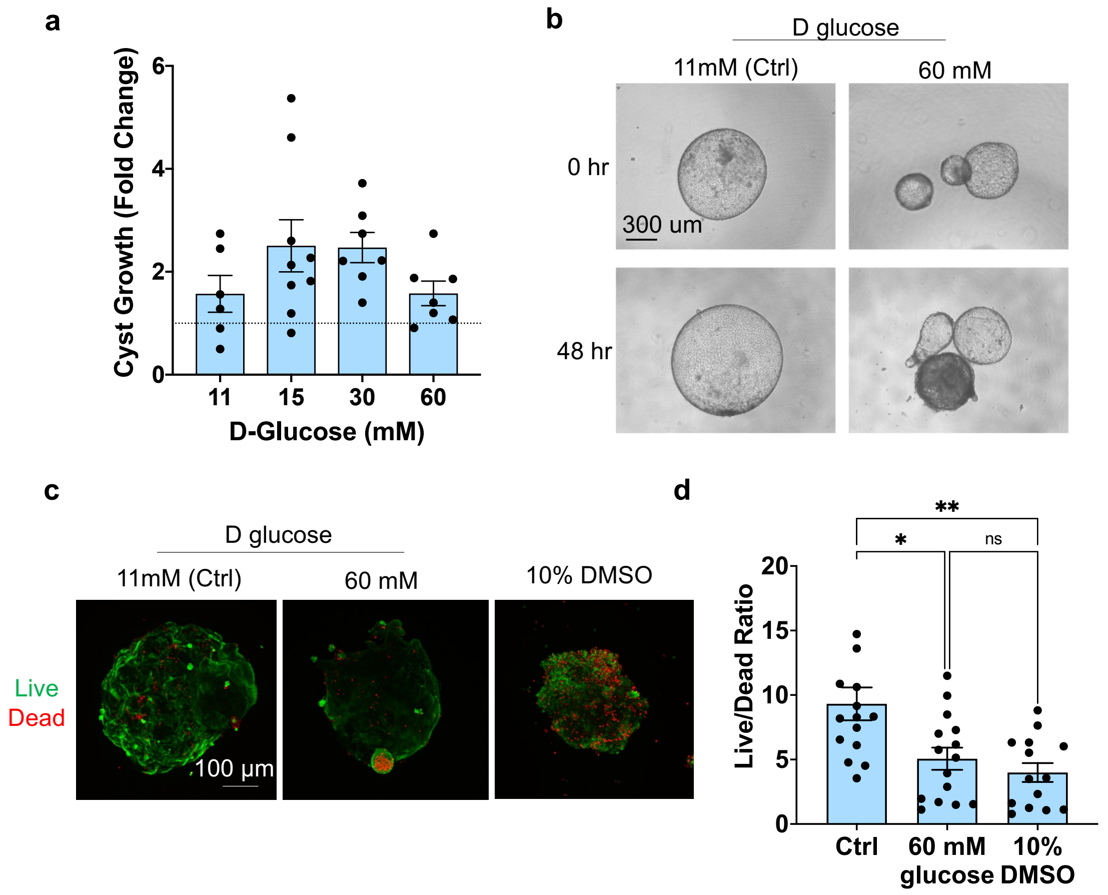

Supplementary Figure 4. (a) Quantification of cyst size fold change for cysts treated with D-Glucose, showing all data points including outliers. **(b)** Representative time lapse phase contrast images of high (60 mM) versus standard (11 mM) glucose treatments. **(c)** Confocal images and **(d)** quantification of live/dead staining (calcein AM/propidium iodide 1:2000) in organoids with high or standard glucose levels, compared to 10 % DMSO as a positive control for cytotoxicity (mean ± s.e.m., n ≥ 14 cysts pooled from 4 independent experiments).

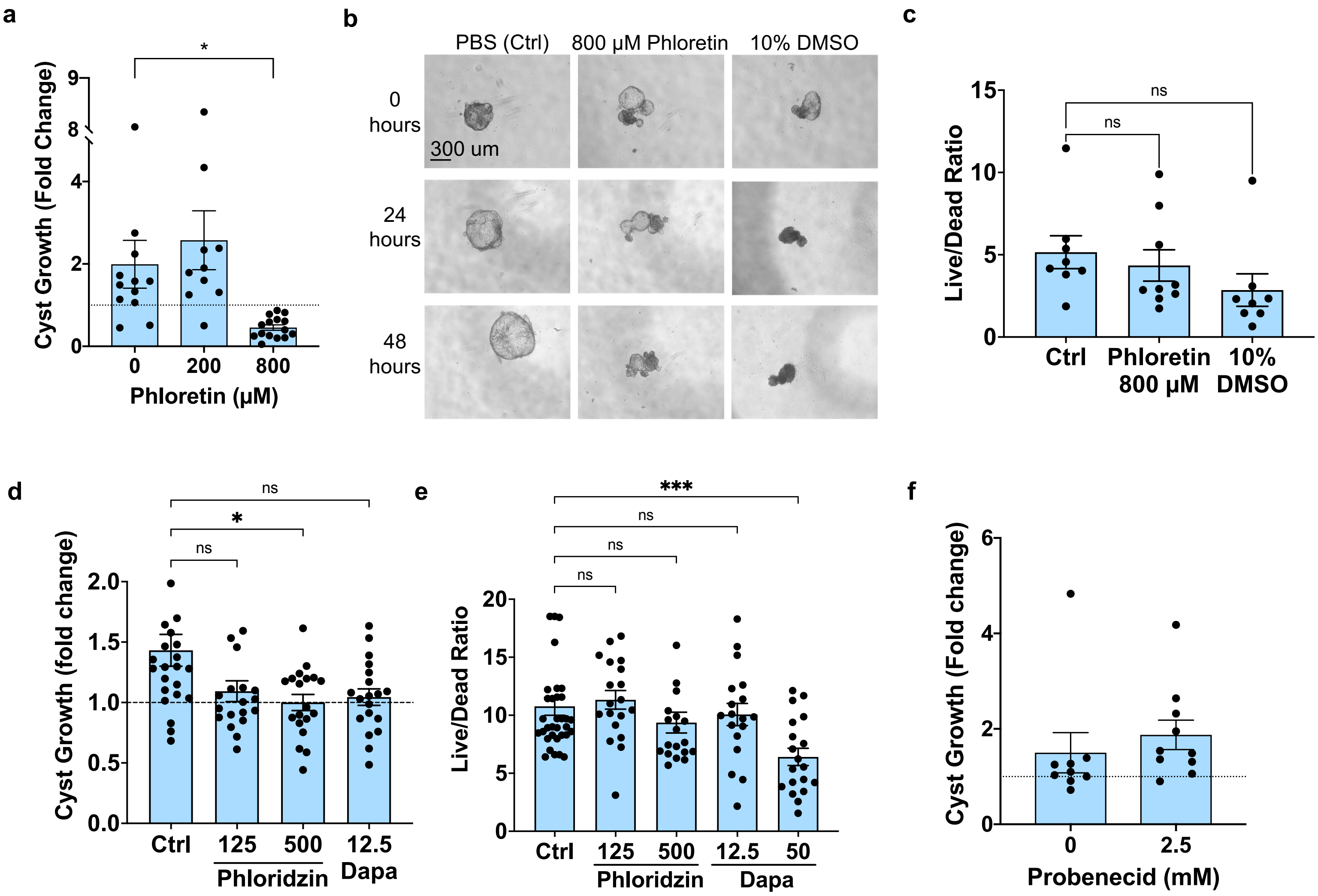

Supplementary Figure 5. (a) Quantification of average change in cyst size for cysts treated with D-Glucose, showing all data points including outliers. **(b)** Representative phase contrast time course images of organoids treated with phloretin or 10 % DMSO, with **(c)** quantification of live/dead staining at 24 hours (n ≥ 7 organoids pooled from 3 independent experiments). **(d)** Quantification of cyst size and **(e)** live/dead ratio for organoids treated for 48 hours with phloridzin and dapagliflozin (mean ± s.e.m., n ≥ 15 cysts per condition, pooled from 3 independent experiments). **(f)** Cyst size quantification of probenecid treatment (n ≥ 9 pooled from 2 independent experiments).

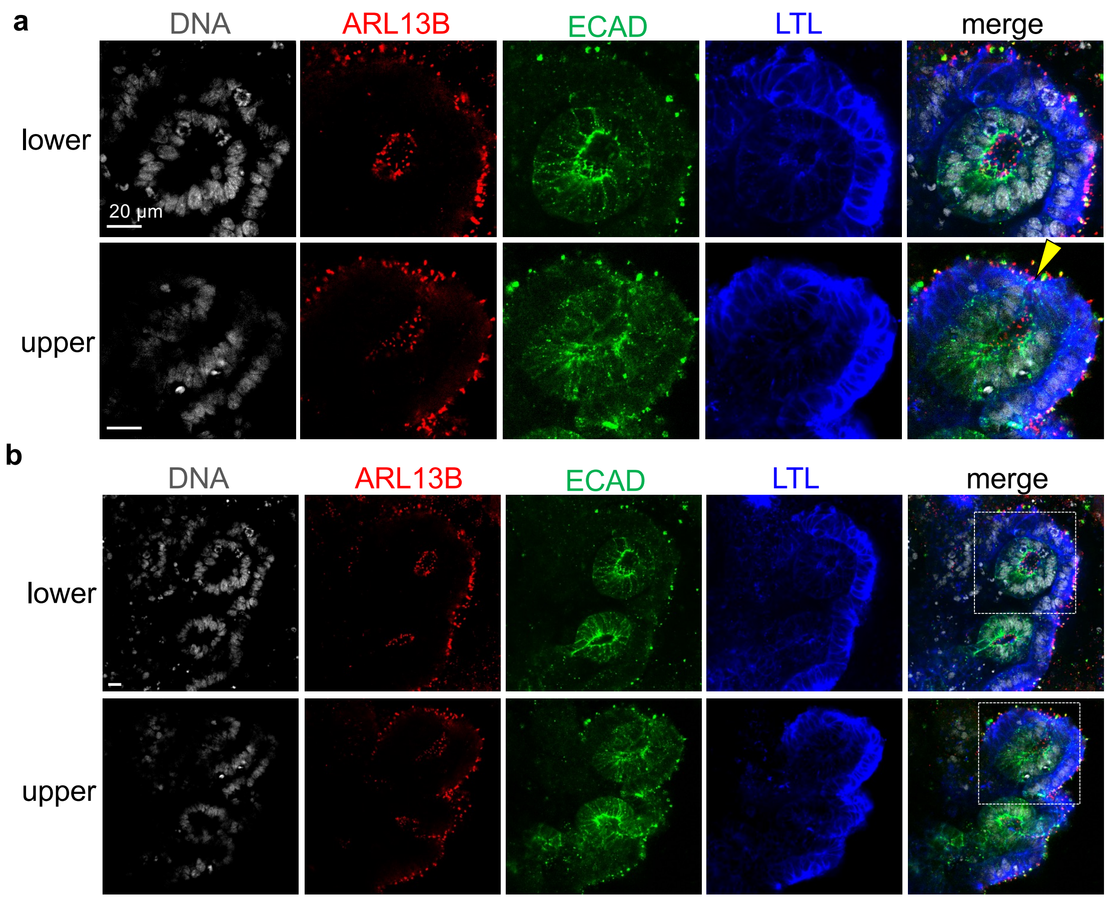

Supplementary Figure 6. Organoid peripheral epithelium faces outwards and contains tubular infolds. (a) Full channel panel and (b) zoomed out confocal immunofluorescence images of control organoids, showing peripheral epithelium. White boxed region highlights the images shown in Figure 5f.

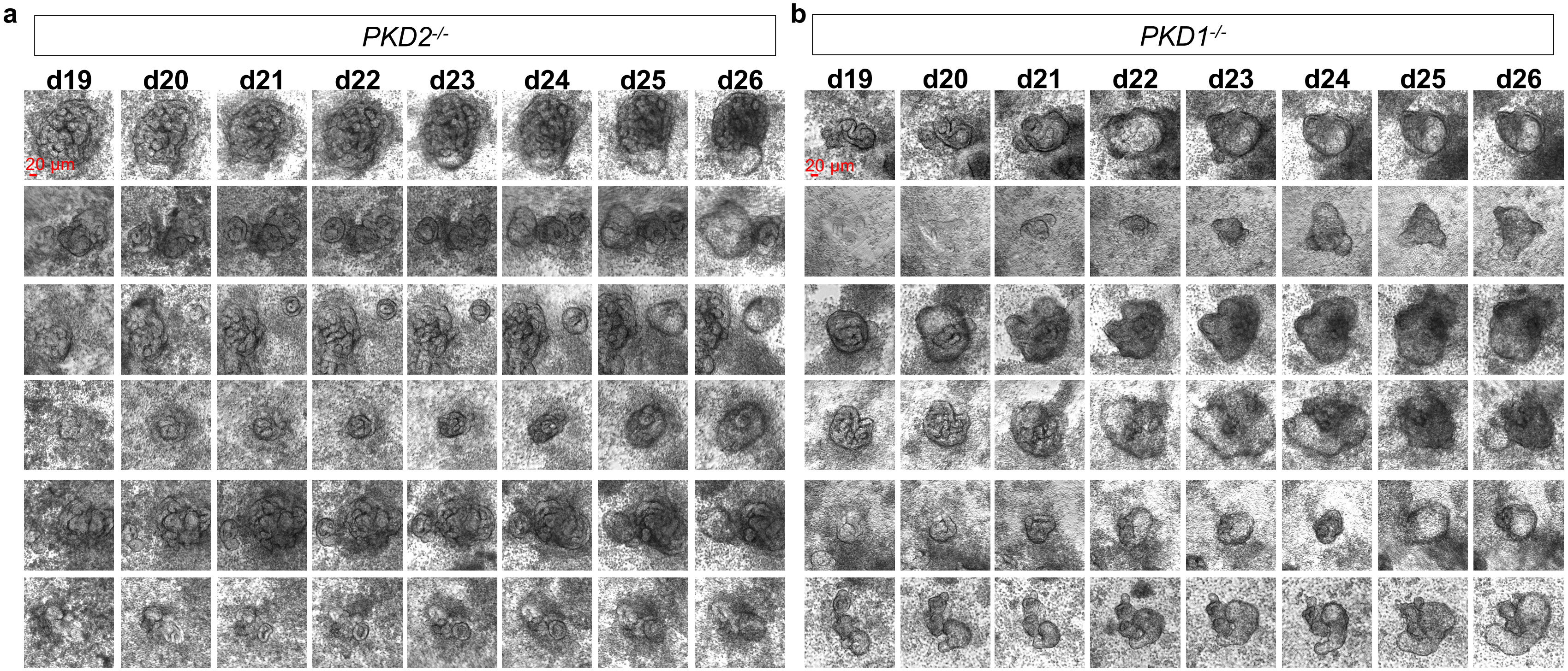

Supplementary Figure 7. PKD organoid cystogenesis occurs via expansion of peripheral epithelium. (a) Time lapse images of cysts forming from 12 representative *PKD2*$^{-/-}$ or **(b)** *PKD1*$^{-/-}$ organoids.

Calculating pressure in 1mL vs. 25mL static conditions

$$\text{Pressure} = \rho\text{gh} = \left(997\,\frac{kg}{m^3}\right)\left(9.81\,\frac{m}{s^2}\right)(x\ m) = Pressure\ \left(\frac{kg}{m \cdot s^2}\right)$$

Height from channel to top of media in reservoir
Static 1mL: ~12 cm
Static 2mL: ~20 cm

$$Pressure_{1mL} = \rho\text{gh} = \left(997\,\frac{kg}{m^3}\right)\left(9.81\,\frac{m}{s^2}\right)(0.12\ m) = 1173.7\ \left(\frac{kg}{m \cdot s^2}\right)\left(\frac{mmHg}{133.32\ Pa}\right) = 8.8\ mmHg$$

$$Pressure_{25mL} = \rho\text{gh} = \left(997\,\frac{kg}{m^3}\right)\left(9.81\,\frac{m}{s^2}\right)(0.20\ m) = 1956.1\ \left(\frac{kg}{m \cdot s^2}\right)\left(\frac{mmHg}{133.32\ Pa}\right) = 14.7\ mmHg$$

Difference in pressure between 1mL and 25mL static conditions = 14.7 – 8.8 = **5.9 mmHg**
