# Supplementary material for: Glucose absorption drives cystogenesis in a human organoid-on-chip model of polycystic kidney disease
Source: Nat Commun. 2022 Dec 23;13:7918. doi: 10.1038/s41467-022-35537-2 (PMC9789147; doi:10.1038/s41467-022-35537-2)
Supplement: Supplementary file 2 — Description of Additional Supplementary Files [file 41467_2022_35537_MOESM2_ESM.pdf]

Description of Additional Supplementary Files:

Supplementary Movie 1. PKD organoids expand under flow. Time-lapse phase contrast images of WTC 11 PKD2-/- vs. control organoids under flow (0.2 dynes/cm2).

Supplementary Movie 2. PKD organoids fail to expand in the absence of diffusion. Time-lapse phase contrast images of WTC 11 PKD2-/- organoid in non-diffusive static condition.

Supplementary Movie 3. A diffusive static condition promotes PKD cyst expansion. Time-lapse phase contrast images of WTC 11 PKD2-/- organoid in diffusive static condition, exposed to 25 mL of culture media.

Supplementary Movie 4. PKD organoids swell rapidly under flow. Time-lapse phase contrast images of WTC 11 PKD2-/- organoid under flow (0.2 dynes/cm2).

Supplementary Movie 5. PKD cysts absorb glucose under flow. Time-lapse phase contrast and GFP channel images of WTC 11 PKD2-/- organoid under flow (0.2 dynes/cm2), 2-NBD Glucose (36.5 µM).

Supplementary Movie 6. PKD cysts absorb glucose under diffusive static conditions. Time-lapse phase contrast and GFP channel images of WTC 11 PKD2-/- organoid in diffusive static condition, exposed to 25 mL of culture media, 2-NBD Glucose (36.5 µM).

Supplementary Movie 7. Glucose is rapidly exchanged in PKD organoids and cysts. Time-lapse phase contrast and GFP channel images of three PKD1-/- organoids in fluidic condition, exposed to 2-NBD Glucose (36.5 µM) for 24 hours, followed by 24 hours of washout with media containing unlabelled glucose without no 2-NBD Glucose.

Supplementary Movie 8. Glucose is rapidly exchanged in a PKD organoid. Time-lapse phase contrast and GFP channel images of PKD2-/- organoid in fluidic condition, exposed to 2-NBD Glucose (36.5 µM) for 24 hours, followed by 24 hours of washout with media containing unlabeled glucose without no 2-NBD Glucose. Phase contrast channel is turned off shortly after glucose addition to highlight absorption (pulse). Phase contrast imaging resumes upon wash-out to record glucose decay (chase).

Supplementary Movie 9. PKD cysts arise from peripheral organoid epithelia. Time lapse phase contrast images of PKD1-/- or PKD2-/- kidney organoids undergoing cyst formation. Four regions of interest are shown for each genotype. Scale is as shown for Figure 5g. Movies are shown first forward in time and then in reverse.
