# Supplementary material for: Glucose absorption drives cystogenesis in a human organoid-on-chip model of polycystic kidney disease
Source: Nat Commun. 2022 Dec 23;13:7918. doi: 10.1038/s41467-022-35537-2 (PMC9789147; doi:10.1038/s41467-022-35537-2)
Supplement: Supplementary file 12 — Reporting Summary [file 41467_2022_35537_MOESM12_ESM.pdf]

nature research

Corresponding author(s): Benjamin Freedman
Last updated by author(s): Dec 4, 2022

Reporting Summary

Nature Research wishes to improve the reproducibility of the work that we publish. This form provides structure for consistency and transparency in reporting. For further information on Nature Research policies, see Authors & Referees and the Editorial Policy Checklist .

Statistics

For all statistical analyses, confirm that the following items are present in the figure legend, table legend, main text, or Methods section.

n/a	Confirmed	
☐	☒	The exact sample size (*n*) for each experimental group/condition, given as a discrete number and unit of measurement
☐	☒	A statement on whether measurements were taken from distinct samples or whether the same sample was measured repeatedly
☐	☒	The statistical test(s) used AND whether they are one- or two-sided<br>*Only common tests should be described solely by name; describe more complex techniques in the Methods section.*
☒	☐	A description of all covariates tested
☒	☐	A description of any assumptions or corrections, such as tests of normality and adjustment for multiple comparisons
☐	☒	A full description of the statistical parameters including central tendency (e.g. means) or other basic estimates (e.g. regression coefficient) AND variation (e.g. standard deviation) or associated estimates of uncertainty (e.g. confidence intervals)
☒	☐	For null hypothesis testing, the test statistic (e.g. *F*, *t*, *r*) with confidence intervals, effect sizes, degrees of freedom and *P* value noted<br>*Give P values as exact values whenever suitable.*
☒	☐	For Bayesian analysis, information on the choice of priors and Markov chain Monte Carlo settings
☒	☐	For hierarchical and complex designs, identification of the appropriate level for tests and full reporting of outcomes
☒	☐	Estimates of effect sizes (e.g. Cohen's *d*, Pearson's *r*), indicating how they were calculated

Our web collection on statistics for biologists contains articles on many of the points above.

Software and code

Policy information about availability of computer code

Data collection	Nikon NIS Elements software was used for image collection, without modification or customization, as indicated in the manuscript.
Data analysis	ImageJ and Nikon NIS Elements software were used for image analysis, without modification, as indicated in the manuscript.

For manuscripts utilizing custom algorithms or software that are central to the research but not yet described in published literature, software must be made available to editors/reviewers. We strongly encourage code deposition in a community repository (e.g. GitHub). See the Nature Research guidelines for submitting code & software for further information.

Data

Policy information about availability of data

All manuscripts must include a data availability statement. This statement should provide the following information, where applicable:

- Accession codes, unique identifiers, or web links for publicly available datasets
- A list of figures that have associated raw data
- A description of any restrictions on data availability

The main data supporting the results in this study are available within the paper and its supplementary information. The raw and analysed datasets generated during the study are too large and complex to be publicly shared (numerous cell lines, replicates, images, blots, and experiments, maintained and analysed in specialized file formats and with unique identifiers). Datapoints are shown as dots in the plots provided in this paper and the Supplement. Source data are provided with this paper. All datasets, including raw data and statistical analysis, are available upon reasonable request from the corresponding author. PKD mutant cell lines used in this study may be obtained from the corresponding author upon request and in accordance with material transfer agreements from the University of Washington and any third-party originating sources.

Field-specific reporting

Please select the one below that is the best fit for your research. If you are not sure, read the appropriate sections before making your selection.

☒ Life sciences ☐ Behavioural & social sciences ☐ Ecological, evolutionary & environmental sciences

For a reference copy of the document with all sections, see nature.com/documents/nr-reporting-summary-flat.pdf

Life sciences study design

All studies must disclose on these points even when the disclosure is negative.

Sample size	Statistical comparisons utilized an unpaired or paired t test (as appropriate for the experiment) for two samples with unequal variance (heteroscedastic, Welch's correction). For multiple comparisons, standard ANOVA test was used. Quantification was performed to measure differences or similarities detectable by eye between separate cultures. Sample size was chosen based on the researchers' qualitative assessment of the reproducibility and variability of each particular experiment (higher variability required greater sample size), and the nature of the data being quantified (whole experiment percentage versus metrics of individual structures).
Data exclusions	Fields and samples for imaging and quantification were chosen at random. Inclusion/exclusion criteria were kept to a minimum and established prior to any quantification.
Replication	All experimental findings were reliably reproduced. All results are representative of multiple biological replicates, meaning either different cell lines with equivalent genotypes were used on a single occasion, or the same cell line was used on different occasions, or both.
Randomization	The experiments were not randomized because they start with separate subclones (mutants). Experimental and control wells (for treatment) and fields and samples (for imaging and quantification) were chosen at random for each experiment. Processing was performed simultaneously and in parallel for all conditions within each experiment.
Blinding	The scientist performing and analyzing the experiments was not blinded to the conditions during the experiments or analysis. However, raw data and results were occasionally shown to blinded investigators, who confirmed the phenotypes.

Reporting for specific materials, systems and methods

We require information from authors about some types of materials, experimental systems and methods used in many studies. Here, indicate whether each material, system or method listed is relevant to your study. If you are not sure if a list item applies to your research, read the appropriate section before selecting a response.

Materials & experimental systems

n/a	Involved in the study	
☐	☒	Antibodies
☐	☒	Eukaryotic cell lines
☒	☐	Palaeontology
☐	☒	Animals and other organisms
☐	☒	Human research participants
☒	☐	Clinical data

Methods

n/a	Involved in the study	
☒	☐	ChIP-seq
☒	☐	Flow cytometry
☒	☐	MRI-based neuroimaging

Antibodies

Antibodies used	Primary antibodies or labels include acetylated alpha-tubulin (Sigma T7451), ZO-1 (Invitrogen 61-7300), Biotinylated LTL (Vector Labs B-1325), E-Cadherin (Abcam ab11512), SGLT2 (Abcam ab37296), laminin-1 (Sigma L9393), alpha smooth muscle actin (Sigma A2547), CD31 (BD Biosciences 557355). 2-NBD-Glucose fluorescent glucose was Abcam ab146200.
Validation	Commercial antibodies were used. In addition to the manufacturer's characterization, many of these have all been validated in our previous papers (Freedman et al., JASN, 2013; Lam, Freedman et al., JASN, 2014; Freedman et al., Nat Comms, 2015; Cruz et al. Nat. Materials, 2017).

Eukaryotic cell lines

Policy information about cell lines

Cell line source(s)	WA09-based cell lines are derived from a female human embryo (originally derived by James Thomson). WTC11-based cell lines are reprogrammed from male skin fibroblasts (originally derived by Bruce Conklin).

Authentication	Cell lines were confirmed as hPSCs based on pluripotency marker expression and differentiation in vitro and in vivo into all three germ layers. These characteristics are unique to hPSCs. Karyotyping confirmed correct gender and the expected chromosome number for each line. In addition, morphological, passaging, and differentiation characteristics of these cell lines matched those expected for WA09 and WTC11 hPSCs.
Mycoplasma contamination	Mycoplasma contamination was tested and found to be negative.
Commonly misidentified lines (See ICLAC register)	No cell lines were used in this way.

Animals and other organisms

Policy information about studies involving animals; ARRIVE guidelines recommended for reporting animal research

Laboratory animals	Pkd1RC/RC mice were obtained from the Mayo Clinic PKD Translational Research Center and bred as heterozygotes crossed with themselves. In order to investigate the process of cystogenesis, younger Pkd1RC/RC mice 6-7 weeks of age, along with wild-type C57BL/6J mice of the same age were used. For glucose perfusion experiments, older animals (> 8 months) were used.
Wild animals	n/a
Field-collected samples	n/a
Ethics oversight	All animal studies were conducted in accordance with all relevant ethical standards under protocols approved by the Institutional Animal Care and Use Committee at the University of Washington in Seattle. Mice were maintained on a standard diet under standard pathogen-free housing conditions, with food and water freely available.

Note that full information on the approval of the study protocol must also be provided in the manuscript.

Human research participants

Policy information about studies involving human research participants

Population characteristics	Participants of any age with polycystic kidney disease
Recruitment	Participants learn of the study through word-of-mouth, the internet, or a letter in the mail. They must contact the PI (Dr. Freedman) to enroll. Participants are consented by a member of the study team. After returning the signed recruitment consent form and questionnaire, they are enrolled in the study.
Ethics oversight	Research complied with all relevant ethical regulations. Human PKD kidney tissue (nephrectomy) was obtained with informed consent under a human subjects protocol approved by the University of Washington Institutional Review Board. No compensation was provided to study participants.

Note that full information on the approval of the study protocol must also be provided in the manuscript.
