# Supplementary material for: Glucose absorption drives cystogenesis in a human organoid-on-chip model of polycystic kidney disease
Source: Nat Commun. 2022 Dec 23;13:7918. doi: 10.1038/s41467-022-35537-2 (PMC9789147; doi:10.1038/s41467-022-35537-2)
Supplement: Supplementary file 13 — Source Data [file 41467_2022_35537_MOESM13_ESM.zip › Source Data/SourceData.pptx]

## Slide 1
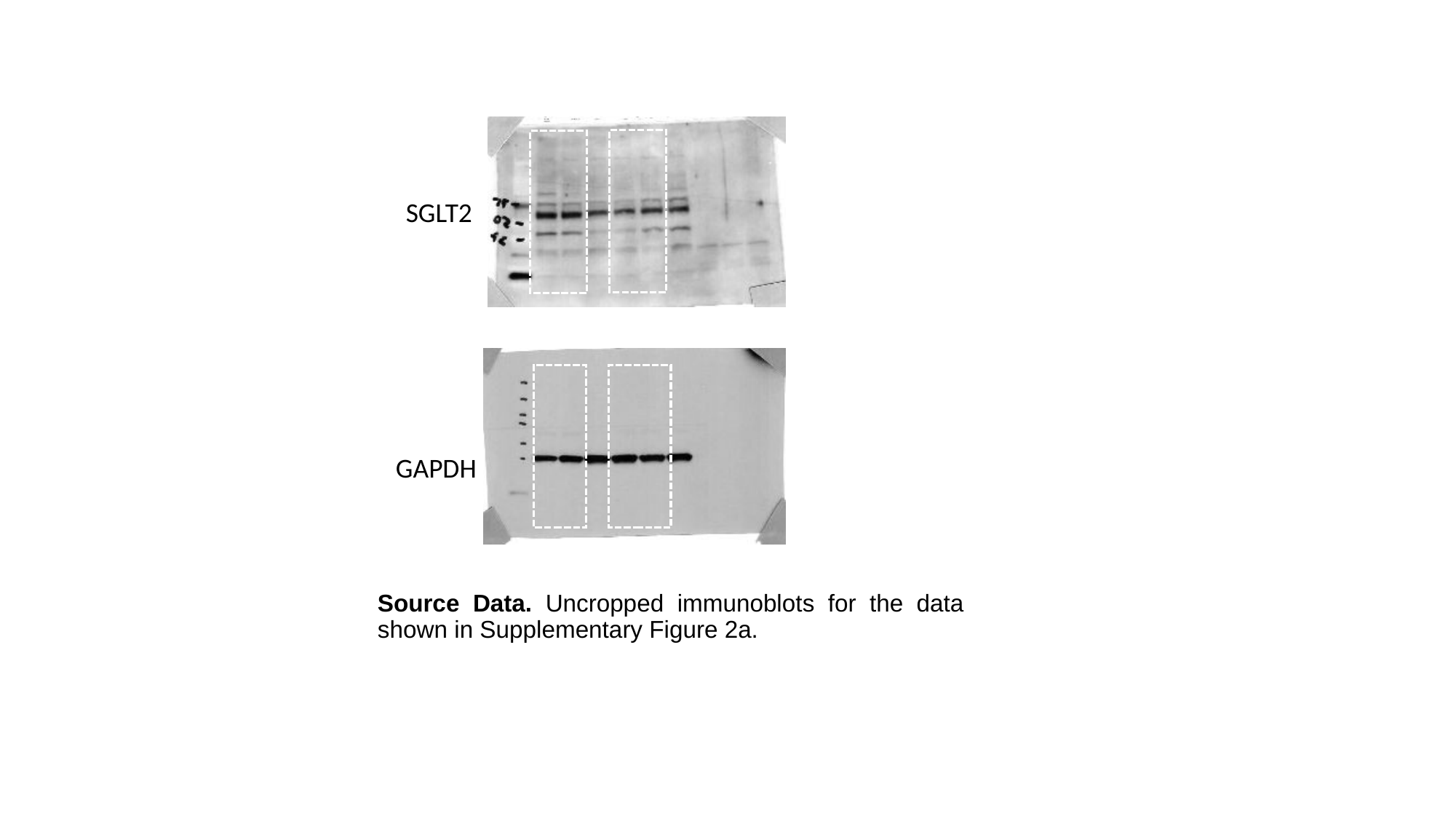

SGLT2
GAPDH
Source Data. Uncropped immunoblots for the data shown in Supplementary Figure 2a.
